# Hydrotherapy and acupressure in restless legs syndrome: A randomized, controlled, 3-armed, explorative clinical trial

**DOI:** 10.1097/MD.0000000000034046

**Published:** 2023-06-30

**Authors:** Julia Kubasch, Miriam Ortiz, Sylvia Binting, Ryan King, Joanna Dietzel, Rainer Nögel, Josef Hummelsberger, Stefan N. Willich, Benno Brinkhaus, Michael Teut, Julia Siewert

**Affiliations:** a Charité – Universitätsmedizin Berlin, corporate member of Freie Universität Berlin and Humboldt-Universität zu Berlin, Institute of Social Medicine, Epidemiology and Health Economics, Berlin, Germany; b International Society for Chinese Medicine (SMS), Munich, Germany.

**Keywords:** acupressure, complementary and integrative medicine, hydrotherapy, Kneipp therapy, randomized controlled trial, restless legs syndrome, study protocol

## Abstract

Restless legs syndrome (RLS) is a common neurological disease that has a significant impact on daily activities and quality of life, for which there is often no satisfactory therapy. Complementary medicine, such as acupressure and hydrotherapy, is used to treat patients with RLS; however, the clinical evidence is unclear. This study aims to investigate the effects and feasibility of self-administered hydrotherapy and acupressure in patients with RLS.

**Methods:**

This is a randomized, controlled, open-label, exploratory, clinical study with 3 parallel arms, comparing both self-applied hydrotherapy (according to the German non-medical naturopath Sebastian Kneipp) and acupressure in addition to routine care in comparison to routine care alone (waiting list control) in patients with RLS. Fifty-one patients with at least moderate restless-legs syndrome will be randomized. Patients in the hydrotherapy group will be trained in the self-application of cold knee/lower leg affusions twice daily for 6 weeks. The acupressure group will be trained in the self-application of 6-point-acupressure therapy once daily for 6 weeks. Both interventions take approximately 20 minutes daily. The 6-week mandatory study intervention phase, which is in addition to the patient preexisting routine care treatment, is followed by a 6-week follow-up phase with optional interventions. The waitlist group will not receive any study intervention in addition to their routine care before the end of week 12. Outcome parameters including RLS-severity, disease and health-related quality of life (RLS-QoL, SF-12), Hospital Anxiety and Depression Score in German version, general self-efficacy scale, and study intervention safety will be measured at baseline and after 6 and 12 weeks. The statistical analyses will be descriptive and exploratory.

**Conclusion:**

In the case of clinically relevant therapeutic effects, feasibility, and therapeutic safety, the results will be the basis for planning a future confirmatory randomized trial and for helping to develop further RLS self-treatment concepts.

## 1. Introduction

### 1.1. Background and rationale

Restless legs syndrome (RLS), also known as Willis-Ekbom disease (WED), is a common neurological disorder characterized by agonizing sensations in the legs, occurrence at rest, and compelling urge to move the legs (or arms or sometimes other body parts).^[1]^ The diagnosis is primarily made by taking a clinical history (essential questions) and can be obtained by any physician, regardless of their specialty. RLS is a common disorder with a significant impact on daily activities and quality of life. Circadian fluctuations in RLS symptoms, with main symptoms in the evening and at night, often cause severe sleep disturbances and deprivation.^[2–4]^

RLS symptoms of any frequency and severity occur in 5% to 10% of the general population in Western industrialized countries.^[5–7]^ Approximately 1 in 4 RLS sufferers is aware of the diagnosis, and overall, 1 in 5 RLS sufferers desire drug treatment to relieve symptoms effectively.^[8]^

RLS affects and interacts with many comorbidities, and may lead to missed work, loss of social networks, and even early retirement.^[9,10]^ The impact of RLS can be very severe, and the quality of life of those affected is generally worse than that of other chronic diseases, such as type 2 diabetes, depression, and osteoarthritis.^[11]^ Although cost studies have shown certain limitations (the use of self-administered questionnaires), the results show that the costs related to RLS are substantial.^[12]^

Patients with RLS also show more anxiety and depression symptoms, psychopathological symptoms, and lower well-being than control subjects without sleep disorders. There is a relationship between RLS severity, anxiety, and depressive symptoms. Under psychological stress, RLS/WED symptoms are more severe.^[13,14]^

RLS is a complex disorder in which predisposing genetic factors, environmental factors, and comorbidities contribute to the expression of the disorder. Patients with a high burden of comorbidity have a consistently higher prevalence of RLS than healthy individuals do. Additionally, an increasing number of publications have reported associations between RLS and multiple diseases, including metabolic diseases (diabetes mellitus and iron deficiency), cardiac and renal diseases, autoimmune diseases (e.g., multiple sclerosis), polyneuropathy, neurodegenerative diseases (e.g., Parkinson disease), and illnesses associated with inflammation and depression.^[2]^

In many cases, RLS remains incurable; therefore, available treatments focus on alleviating the symptoms of the disease.^[2]^ However, the side effects of currently used medications, such as dopaminergic agents and non-ergot- and ergot-derived dopamine agonists, may lead to other health problems. The most important side effect is the so-called augmentation, that is, the amplification of RLS symptoms, which occurs in 30% to 68% of patients taking dopaminergic drugs.^[15]^ Other typical side effects of dopamine agonists, especially during the first few weeks of treatment, include edema, nausea, orthostatic dysregulation, dizziness, and lightheadedness.^[16]^

Dopamine agonists may also lead to impulse control disorders in up to 12.4% of patients taking the dopamine agonist Pramipexole).^[17]^ Other non-dopaminergic drug classes for the treatment of RLS, such as opioids, anticonvulsants, and alpha-adrenergic agonists (Clonidine), also have typical and well-known side effects, such as dizziness, somnolence, tremor, headache, mental changes, nausea, vomiting, urinary retention, and constipation, which often lead to therapeutic limitations. The addictive potential of opioids is another therapy-limiting and health-threatening issue.^[18–22]^

Effective non-drug symptom reduction in RLS would be clinically helpful and relevant because RLS-medications with potential side effects, such as augmentation, could be avoided or reduced. State of the art reviews on RLS^[23]^ recommend that all non-drug treatment options be exhausted before starting drug treatment. According to the guidelines of the International Group Task Force, increases in medication over the course of treatment should be limited to the onset of clinically important symptoms that cannot be managed behaviorally.^[24]^

For the treatment of mild and intermittent forms of RLS, clinical guidelines often recommend relaxation methods and other non-pharmacological therapies.^[24–26]^ Recommendations are generally nonspecific and there is little meaningful research evidence.^[27,28]^

Up to 65% of RLS patients regularly use complementary medical interventions to relieve their symptoms and improve well-being.^[29]^

Hydrotherapy according to Kneipp and acupressure are methods of complementary and integrative medicine that can be performed by the patients themselves and at a low cost. To date, no randomized controlled trials have examined the effectiveness, safety, and feasibility of self-administered acupressure and hydrotherapy according to Kneipp in patients with RLS.

Two systematic reviews in 2019 and 2021 showed that acupuncture was significantly superior to control interventions for RLS in terms of severity and reported significant clinical effects.^[27,30–32]^ While acupuncture requires a trained therapist to stimulate specific acupoints with needles, acupressure involves massaging the same acupoints with fingers or hands, making it suitable for self-application.

In a pilot study on the efficacy of acupressure in dialysis patients with RLS, a reduction in RLS severity was reported.^[33]^ Several studies have demonstrated that acupressure can improve sleep-related symptoms, depression, anxiety, stress, pain, and psychological distress.^[34–40]^

Hydrotherapy according to Sebastian Kneipp (1821–1897) is characterized by serial, mostly cold-water applications (e.g., affusions, compresses, washes) and has been known in German-speaking countries since the 19^th^ century for preventive health care and the treatment of various diseases.^[41]^

There are indications that cold water applications can reduce RLS symptoms in pregnant women,^[42]^ and cold applications in general can produce an overall reduction in sleep-related symptomatology.^[27,30,32]^ Kneipp therapy showed significant improvements in sleep quality and well-being in non-organic sleep disorders in a controlled study.

Self-administered acupressure and hydrotherapy, according to the German non-medical naturopath and priest Sebastian Kneipp, showed little to no side effects.^[35,36,43–45]^

As there is good evidence for the effectiveness of acupuncture in patients with RLS, and some pilot studies have shown that acupressure and cold applications can reduce RLS- and sleep-related symptoms, we hypothesized that self-administered acupressure and Kneipp hydrotherapy with cold affusions could be a potential therapeutic option for patients with RLS.

### 1.2. Study aims

The aim of this clinical study is to assess the feasibility and effects of self-administered acupressure and Kneipp hydrotherapy in patients with RLS, and to gather preliminary information on both study interventions as a basis for conducting a high-quality confirmatory trial including a valid sample size calculation.

## 2. Methods

### 2.1. Study design

**Figure 1.**
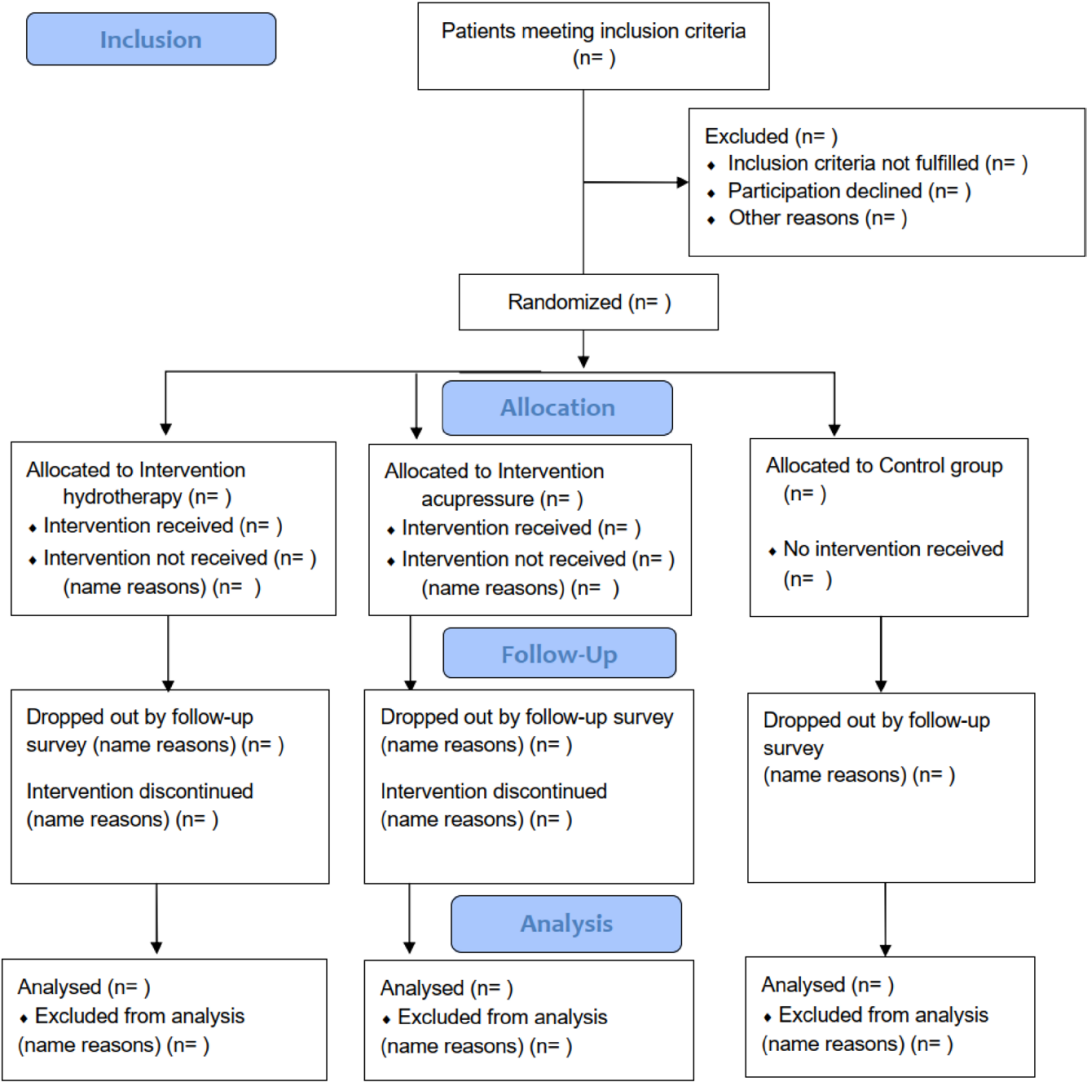
Design of the HYDRAC study. HYDRAC = Hydrotherapy and Acupressure for Restless legs syndrome.

The HYDRAC study (HYDRotherapy and ACupressure for Restless legs syndrome) is a prospective, parallel, 3-armed, interventional, exploratory clinical study. The study will be conducted for 12 weeks per patient, including a self-treatment phase of 6 weeks and a follow-up phase for another 6 weeks with optional self-treatment (Fig. 1).

Patients will be enrolled at the Charité Outpatient Clinic for Complementary and Integrative Medicine in Berlin-Mitte. After training on how to self-perform the respective interventions, the study interventions are to be carried out independently by the patients at home.

To ensure high-quality evidence, we will conduct this study in accordance with Standard Protocol Items: Recommendations for Interventional Trials (Table 1)^[46]^ and report our findings in accordance with the Consolidated Standards of Reporting Trial (CONSORT) Statement.^[47]^ This clinical trial was approved by the Charité Ethics Committee on 19.07.2022 (reference number: EA2/132/22) and has been registered with the German Clinical Trials Registry with an identifier (DRKS00029960) on August 09, 2022.

**Table 1:**
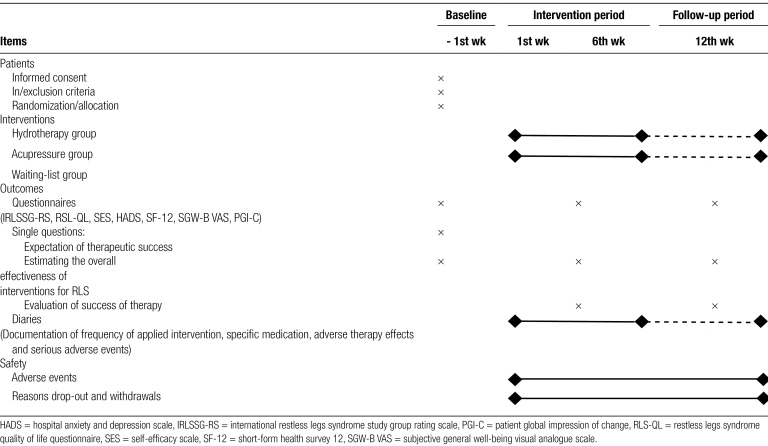
A standard protocol items: recommendation for interventions for trials (SPIRIT).

### 2.2. Patients and recruitment

A total of 51 patients (n = 17 in each arm) included in the trial. Patients who meet the following inclusion criteria will be included in the study: age between 18 and 75 years; diagnosis of RLS confirmed by a trained medical specialist (“Facharzt”) in accordance with the diagnostic criteria,^[48]^ subjective information on the intensity of RLS symptoms greater than or equal to 30 on a visual analog scale of 0 to 100 mm in the last 7 days before inclusion; at least moderate RLS symptoms (IRLS - total score ≥ 11 according to a severity scale validated by the International Restless Legs Syndrome study group (IRLS); no planned change in medication during the study; willingness of the patient to be randomized and to participate in the examinations of the study or to fill in questionnaires/diaries; patient consent; ability to comprehend the significance and content of the study; and presence of a written informed consent form.

Patients who meet any of the following criteria are excluded: indication for iron substitution therapy (exceptions are iron substitution that has already been carried out without sufficient symptom improvement or refusal of the iron substitution therapy by the patient); regular use of RLS triggering/exacerbating medications (such as mirtazapine, mianserin, clozapine, olanzapine, risperidone, haloperidol, sulpiride, promethazine); use of hydrotherapy, acupuncture, or acupressure in the last 4 weeks prior to inclusion or planned in the next 12 weeks; acute infection with severe acute respiratory syndrome coronavirus type 2 virus or presence of long-coronavirus disease syndrome; for women: pregnant or breastfeeding; presence of a serious acute and/or chronic organic or serious mental illness that does not allow participation in the study intervention (e.g., advanced cardio/pulmonary disease New York Heart Association classification/COLD III + IV); known Raynaud disease and advanced circulatory disorders of the extremities; inadequately treated dermatological disease in the therapeutic area (large wounds, severe atopic dermatitis, severe psoriasis, etc); abuse of medicines, drugs and/or alcohol; existing treatment with opioids; participation in an intervention study during the same period of the study or in the last 3 months prior to the start of the study for this condition; dependence on the study site (e.g., employee).

The recruitment of study patients will be carried out primarily by advertising on public transport, newspaper advertisements, newsletters of medical institutions, the internet, flyers at general practitioners’ clinics, and neurological specialist practices. If patients meet the study criteria, they must sign an informed consent form before participating in the study or before randomization. The study physician obtains informed consent and signs a written informed consent form. The consent form contains the agreement to participate in the study, as well as the collection and storage of study data and personal data of the patient.

### 2.3. Study physicians

Both study physicians (JS and JK) fulfill the following requirements: knowledge of data protection, ICH-good clinical practice guidelines, content and procedure of the study, and instructions for the study interventions. They received detailed instruction and supervision by long-standing experts in hydrotherapy according to Kneipp and acupuncture/acupressure. The study physicians all had specialist medical training (German: “Facharztausbildung”) with at least 10 years of professional experience.

### 2.4. Study interventions

#### 2.4.1. Hydrotherapy according to Kneipp

**Figure 2.**
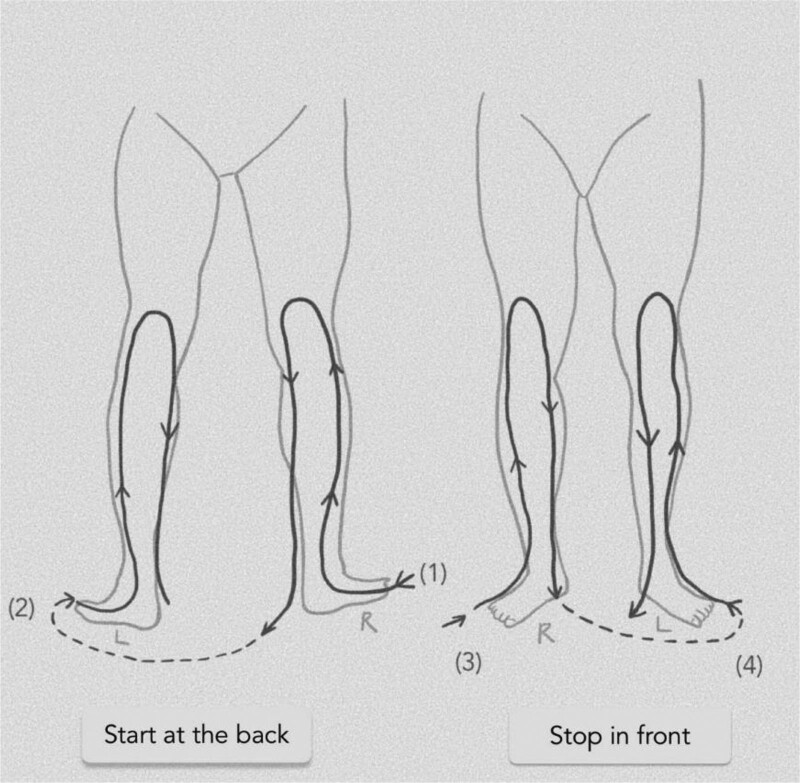
HYDRAC study: Scheme of the study intervention hydrotherapy according to Kneipp. HYDRAC = Hydrotherapy and Acupressure for Restless legs syndrome.

Patients in the hydrotherapy group will be trained by the study physicians on the day of randomization and will receive an illustrated manual. Hydrotherapy should be performed semi-standardized with obligatory and optional affusions. The patients perform the treatments twice daily for 6 weeks. After week 6, further treatment is optional. Recommended affusions are 2 cold affusions up to the knees daily for 30 to 60 seconds or 3 to 6 minutes if performed as alternating warm/cold affusions. (Fig. 2) In total, this takes approximately 20 minutes daily, including preparation and post-processing time. Optional affusions include cold or alternating warm arms and cold face affusions. The optional affusions are selected by a study physician according to the physical condition and symptomatology during training on the day of randomization. Arm affusions can be used in cases of RLS symptoms in the arms, and face affusions can be used in cases of fatigue. Facultative affusions can be performed as often as desired during the day, following the basic Kneipp rules (Table 2). One and 3 weeks after the start of intervention, patients will be contacted by a study physician by phone to inquire about difficulties with implementation, to clarify any questions that have arisen, and to improve adherence to interventions. If necessary, interventions will be adjusted in terms of the application temperature.

**Table 2:** General rules for hydrotherapy applications according to Kneipp (as handed out to patients).

Empirical experience indicates that the success of water treatments depends on the regular and correct way of performing them. The following basic rules should be observed:
•Do not perform the applications in cold or drafty rooms. •Do not perform cold applications on cold body/parts of the body; warm up cold hands and feet before watering, e.g., by gymnastics or a walk or even a warm bath. •After the cold applications the water should not be dried off, if possible, but only wiped off with the hands or carefully dabbed dry. Only the spaces between the toes are dried. •A few minutes after the cold-water application, the body should be completely warmed up again, so please put leggings on immediately after the applications and/or ensure good rewarming through movement or bed rest; do not remain motionless or without clothing/blanket. •Do not apply immediately before or after eating (interval approx. 30 min). •Do not smoke before, after and during the treatment; coffee, tea or alcoholic beverages also impair the reactivity of the capillary vessels and thus also the desired positive reaction. •In case of varicose veins, the water temperature of the warm application should not exceed 37°C (bath thermometer!). •A bluish discoloration of the skin and/or a cold pain are signs of cold application for too long and should be avoided. Please perform the applications only as long as it is comfortable for you. •Cold casts can be intensified by extending the watering time or repeating the affusion (e.g., arm affusion).
When should you not perform hydrotherapy treatments?
•Women should only perform applications on the upper body during menstruation. •Do not perform a knee cast during acute inflammation of the urinary tract. •In case of colds, sinusitis, and trigeminal neuralgia, do not perform facial watering. •In case of open injuries or ulcers of the body parts to be watered.

°C = degree celsius.

#### 2.4.2. Acupressure

**Figure 3.**
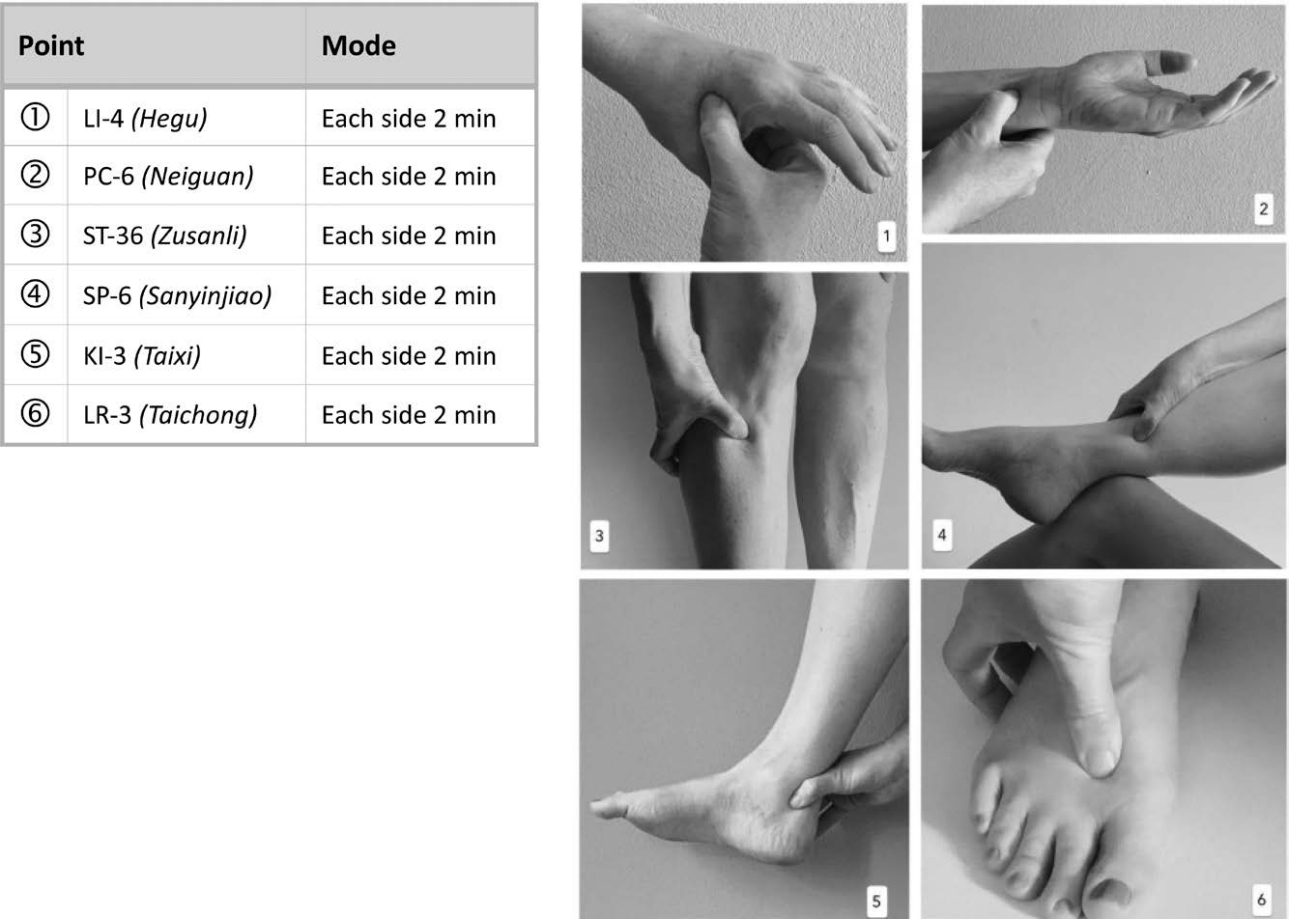
HYDRAC study: Scheme of the study intervention acupressure. HYDRAC = Hydrotherapy and Acupressure for Restless legs syndrome.

Patients in the acupressure group will be trained by the study physicians after inclusion and randomization, and will also receive an illustrated manual. Acupressure should be performed in a standardized manner with a 6 acupuncture point set according to the rules and principles of Chinese Medicine,^[49]^ as well as following a modified expert consensus process. The following acupressure points are used bilaterally: Liver 3 (Liv-3), Spleen 6 (SP-6), Kidney 3 (KI-3), Stomach 36 (St-36), Pericardium 6 (PC-6) and Large Intestine 4 (LI-4) (Fig. 3). The patients carry out the treatments at least once a day for 6 weeks with a total treatment time of approximately 20 minutes daily and a duration per pressure point of approximately 2 minutes. The points can be pressed simultaneously on both sides if the patient wishes. One and 3 weeks after the start of the intervention, patients will be contacted by telephone to inquire about difficulties in practice, answer any questions that may have arisen, and improve their adherence to the intervention. If necessary, intervention (number of acupressure points) will be adjusted. After week 6, further treatment is at the patient discretion.

#### 2.4.3. Waiting list group

Patients in the waitlist group continue to take their routine medication and will not receive any additional training during the intervention and follow-up periods. They have the option to receive free hydrotherapy or acupressure training by one of the study physicians after completing the study.

Medically necessary treatments during the study are allowed for all groups. Changes in RLS medication are recorded in patients’ diaries.

#### 2.4.4. Outcomes

Patients will receive the following questionnaires, which will be completed at baseline and 6 and 12 weeks after the start of the intervention phase:

*International Restless Legs Syndrome Study Group Rating Scale for Severity of Restless Legs Syndrome*^[50]^: 1 of 3 assessments which is validated for the German language area for the assessment of RLS severity and for the characterization of RLS

*Restless Legs Syndrome Quality of Life Questionnaire (RLS-QoL*)^[51]^: An 18-item scale to assess the quality of life of patients with restless legs syndrome.

*Quality of Life Short Form 12*^[52,53]^: general health questionnaire, which refers to 8 different dimensions that allow a statement about the health status of the patients.

*Hospital Anxiety and Depression Scale*^[54]^: assesses depression and anxiety severity in patients with somatic diseases or (possibly psychogenic) physical complaints.

*General Self-Efficacy Scale*^[55,56]^: A self-assessment procedure with ten items for recording general optimistic self-beliefs.

*Visual Analogue Scale*^[57]^: measures subjective general well-being on a visual analog scale (minimum 0 to maximum 100 mm).

*Patient Global Impressions Scale—Change*^[58]^ evaluates all aspects of the patient health status and assesses whether there has been improvement or deterioration in clinical condition.

We also want to investigate whether patients perceive a change in daily activities and how they estimate the effectiveness of the intervention before randomization, the success of the therapy, and the overall effectiveness of the interventions at the end of weeks 6 and 12.

In addition, the patients keep a diary to record the frequency of the study interventions and changes in their medication daily from week 1 to week 6. In the diary, patients will also note any side effects, adverse events, or serious adverse events. The first diary, which is handed out to the patients after randomization, is kept daily from weeks 1 to 6. The second diary is mailed to patients 12 weeks after enrollment, that is, at the end of the follow-up period, and is intended to retrospectively record the last 6 weeks of voluntary intervention, that is, safety, medication use, and continuation/frequency of intervention.

#### 2.4.5. Blinding procedures

Due to the nature of Kneipp hydrotherapy and acupressure, patients and study physicians cannot be blinded. To minimize bias, all outcomes at baseline and 6 and 12 weeks after randomization will be evaluated by the same independent, experienced investigators blinded to the group assignment. After the completion of the statistical analyses, the group assignment will be announced by the research associate for biometrics.

#### 2.4.6. Randomization

Randomization will be performed centrally based on a computer-generated randomization list (generated using R software (version 4.1.2)) and will be block randomization with variable block length. Allocation to the 3 groups will be in a 1:1:1 ratio. Randomization will be performed at the end of the inclusion examination by the study physician using an administrative database. This will happen after informing about the study, providing written informed consent, and reviewing the inclusion and exclusion criteria. The last name, first name, date of birth, and sex will be entered into the administrative database by the study physician. The system performs automated randomization, and randomization confirmation is generated and printed using a button click. All further personal data will then be completed by the study nurse. The result of the randomization is communicated to the patients when the baseline questionnaires have been completed. Subsequently, training in the respective interventions will take place.

### 2.5. Sample size calculation

As this is an exploratory study to generate the first results and further queries about the effect of hydrotherapy and acupressure in patients with RLS, we did not make a sample size estimation. As we expect about 10% of the patients to drop out of the study before week 12; 17 randomized patients per group (51 randomized patients in total) are planned and seem logistically feasible at the study center. Thus, we expect to have at least 15 patients aged 18 to 75 years per group at week 6.

### 2.6. Attendance and drop-outs

The attendance of each patient is recorded by the return and evaluation of diaries and questionnaires. Dropouts and reasons for dropout will be recorded in the study database.

### 2.7. Statistical methods

All data collected are analyzed descriptively: means, standard deviation, median and quartile, or frequencies and percentages (overall and separated by intervention group). Endpoints are analyzed graphically and in an exploratory manner, and depending on the scale, performed using analysis of covariance or logistic regression (in each case, the treatment group and (if available and in case of relevant group differences) the respective baseline values are adjusted for as covariates). Adjusted means or odds ratios with 95% confidence intervals and p-values for the group comparisons are reported. All *P* values are considered exploratory without adjustment for multiple testing. Analyses are performed using the full analysis set, and the population is defined according to the intention-to-treat principle, that is, classification according to each randomization group, for patients with the data available for the respective evaluation. There will be no substitution for the missing data. Analyses are performed using statistical package for social sciences (SPSS) (IBM SPSS Statistics, version 25), R (version 4.1.2), and SAS (SAS for Windows, version 9.4).

### 2.8. Data collection and management

The personally identifiable data are entered and managed in a password-protected MS Access database and serve as a re-identification list. The database resides on a separate project drive, with limited accessibility. Data from the survey instruments are entered in pseudonymized form into a SoSci-Survey online database programmed by the Institute of Social Medicine, Epidemiology, and Health Economics at Charité Universitätsmedizin Berlin, via an HTTPS connection (SSL encryption) with an existing Internet connection to the server, which is located within the Charité IT infrastructure. The data received from the server can be exported for further processing, and this can only be performed by the data manager via a password-protected login. The exported data are stored on a specially protected drive within the server of the Institute of Social Medicine, Epidemiology, and Health Economics.

The questionnaires are entered into the online database in a pseudonymous form by 1 or more persons known by name and authorized by the principal investigator. The entry of the questionnaires is also password protected. After checking for correctness and plausibility, the data are transferred to SPSS data format.

### 2.9. Withdrawal criteria and management

Patients may or must withdraw from the study if the continuation of the study imposes an unreasonable burden on the patient because of the patient condition, if a serious adverse event occurs and a causal relationship is established by the study director, if patients request withdrawal from the study and/or there is a lack of willingness to cooperate or comply, and/or if circumstances arise that make continued participation unreasonable, such as massive deterioration in health due to serious illness.

Reasons and timing of withdrawal will be recorded in standard case report forms and stored in the study database.

### 2.10. Oversight and monitoring

#### 2.10.1. Safety monitoring

Together with the study documents, all patients will receive the necessary contact information to report any serious events directly to the Institute of Social Medicine, Epidemiology and Health. Serious Adverse Events must be reported by the study physicians to the study management within 24 hours of being known.

#### 2.10.2. Dissemination plans

The authors intend to publish the results of this study in peer-reviewed journals and present them at local and international conferences. A summary of the results can be provided to the patients who participated at their request.

## 3. Discussion

The present exploratory RCT will be the first to investigate whether there is evidence for the effects of self-applied Kneipp hydrotherapy and acupressure in patients with RLS and whether it is reasonable to conduct a subsequent confirmatory study in a larger patient population.

Our requirement is that the inclusion and exclusion criteria be comparable to those of other high-quality studies on RLS. Thus, the study inclusion and exclusion criteria were based on the RLS/WED diagnostic criteria according to the updated International Restless Legs Syndrome Study Group (IRLSSG) consensus criteria of 2014.^[48]^ The age criterion was initially set at 18 to 70 years, considering the capacity for consent and exercise. It increased to 18 to 75 years in an amendment due to several requests from patients over 70 years old. In this amendment, the exclusion criteria were also adjusted due to an update of the national RLS guideline,^[26]^ as a distinction between primary and secondary RLS was removed; thus, all patients with confirmed RLS were included in the study. Previously, the exclusion of patients with secondary RLS was planned. In exchange, we expanded the exclusion criteria to include the need for iron supplementation unless contraindications were present, or supplementation was unsuccessful. Other inclusion and exclusion criteria were contraindications to the use of interventions, such as open wounds in the intervention area or severe cardio/pulmonary disease (New York Heart Association classification/global initiative for chronic obstructive lung disease III + IV).

We want to investigate not only a non-drug approach but also an intervention that can be performed independently by the patient. Self-applicable therapies empower patients, but rely on motivation. To promote motivation and adherence to the intervention, we use diaries and contact patients by telephone 1 and 3 weeks after randomization. Problems regarding implementation and the need for further training will be ascertained through telephone interviews. This also allows for immediate optimization of the intervention and (if necessary) improves the implementation of the intervention and the intervention itself in a future full-scale study.

All patients in the intervention groups also receive a handout with the corresponding intervention instructions in the text and pictures at the beginning of the study.

This study uses a well-defined acupressure protocol that focuses on 6 specific acupuncture points, some of which have already been shown to be effective in previous acupuncture and acupressure studies in RLS^[30,31,33]^ and were finally determined by consensus by our own experts in Chinese medicine with more than 25 years of acupuncture practice. A balance was reached between a sufficiently large number of acupoints to achieve an effect and a small number of points so that the daily time commitment for the patients remains manageable and feasible. The strength of the pressure is demonstrated during the instruction and should trigger a sensation just below the pain threshold.

Kneipp hydrotherapy was chosen because it is a well-known and recognized treatment concept in Germany that can be carried out independently at home without additional equipment and without much time expenditure, and has shown good acceptance in previous studies.^[45]^ We chose cold knee affusion as a mandatory application because it is easy to perform and is considered the most affected body region of RLS patients. Among other modes of action, cold-water hydrotherapy works by stimulating the sympathetic nervous system, whereby the sympathetic tone is lowered over time as part of an adaptation process.^[45,59]^ In addition, the cold stimulus provokes local hyperemia, and thus muscular relaxation and stimulation of local metabolic processes. Since several publications on the etiology of RLS suggest the involvement of the autonomic nervous system and peripheral tissue hypoxia,^[60–62]^ it is assumed that hydrotherapy according to Kneipp could be beneficial for patients with RLS owing to the effects mentioned above.

A twice-daily application was chosen because a previous study on Kneipp hydrotherapy in patients with menopausal symptoms showed^[45]^ that this is a feasible and acceptable frequency of application, and that greater effects can be expected than with a once-daily application. As this is an exploratory study, patients are free to perform the treatments more frequently, both acupressure and hydrotherapy. For both groups, there should be an approximately equal time commitment of 20 minutes daily.

By recruiting patients broadly, both online, in public transportation, and in physicians’ offices and through self-help groups, the study aims to find a representative group of patients in terms of socioeconomic status, age distribution, and comorbidities. This study uses valid, reliable, and internationally accepted assessment tools to measure changes in RLS-severity, including sleep quality, depression, anxiety, and QoL. If effects are detectable, the data obtained can help determine recruitment potential, subsequent sample size calculation, reasonable number and frequency of interventions, and further parameters needed to successfully design a subsequent full-scale study.

### 3.1. Strength and limitations of the study

This study is the first to investigate 2 non-drug therapy options for RLS that could expand the current treatment spectrum through self-applicable, openly accessible, and cost-effective measures. On the one hand, both therapies can be implemented in addition to the existing routine therapy, and, on the other hand, they can potentially limit or prevent the side effects of the existing drug therapy by helping to reduce the dose. After instruction, patients are largely independent of therapists in the implementation of the intervention and gain self-efficacy in the best case.

Limitations arise from the fact that this is an exploratory study designed to investigate feasibility and assess potential impact, but not a study with a design that allows the confirmation of a formal hypothesis, a primary outcome based on a valid sample size calculation. In this study, routine care alone is the control condition. This means that patients assigned to the control group do not receive additional intervention in their routine care, as is the case with the hydrotherapy and acupressure groups. This means that there is no comparison group for possible specific effects of acupressure or hydrotherapy, such as sham procedures, on both study interventions. Furthermore, the study physicians and patients are not blinded to the interventions, whereas the data analysts are. Therefore, if there are clinically relevant effects of the interventions, we cannot say that they are due to them. It could also be due to other factors, such as “doing something,” “taking time for oneself,” “relaxing,” and “participating in a study.” Patients in the control group could be frustrated about being assigned to a “waiting group” and could be influenced in their outcome evaluation by negative affect.

However, self-applied acupressure and hydrotherapy are not easily blinded, making the sham control of the 2 study interventions difficult.

Because outcome parameters are collected solely from patients and through subjective measures, behavioral change and dissatisfaction may occur if patients do not receive the desired intervention. These patients are also more likely to be lost to follow-up.

Another critical point could be the training of the patients. Live training for the study interventions will occur only once at the beginning of the intervention, and there will be no personal visual inspection by the study physicians during the study to ensure that the applications are performed correctly. This can lead to a situation in which the possible effects of the interventions do not take effect because they were performed incorrectly. Therefore, in the case of missing effects, it is not possible to say with certainty whether this is due to incorrect execution or the lack of effect of the study intervention itself.

Moreover, patients in the acupressure group have some freedom in choosing the quality of the pressure to be applied (e.g., constant pressure, pulsating massage), which could lead to different results according to the theory of Chinese medicine.

This exploratory study can provide a better understanding of the effects of complementary and self-administered interventions in patients with RLS. This may bring new questions to light so that future research questions in the field of RLS therapy can be refined. This study can also be used to formulate hypotheses about the causal relationships between the study interventions and their impact on RLS severity and quality of life in patients with RLS. Another advantage of this study is that we can see how both interventions work in their natural environment, what problems occur, and whether it is safe to do them at home.

## Acknowledgments

We would like to thank the entire study team, including Margit Cree and Katharina Kleinsteuber (members of the HYDRAC study secretary) for their outstanding work on this study.

## Author contributions

**Conceptualization:** Miriam Ortiz, Joanna Dietzel, Rainer Nögel, Josef Hummelsberger, Benno Brinkhaus, Michael Teut, Julia Siewert, Julia Kubasch.

**Data curation:** Sylvia Binting, Ryan King, Julia Siewert.

**Funding acquisition:** Benno Brinkhaus, Michael Teut, Julia Siewert.

**Investigation:** Julia Kubasch, Benno Brinkhaus, Michael Teut, Julia Siewert.

**Methodology:** Sylvia Binting, Ryan King, Benno Brinkhaus, Michael Teut, Julia Siewert.

**Project administration:** Julia Siewert.

**Supervision:** Benno Brinkhaus, Michael Teut, Julia Siewert.

**Writing – original draft:** Julia Kubasch, Julia Siewert.

**Writing – review & editing:** Julia Kubasch, Miriam Ortiz, Sylvia Binting, Joanna Dietzel, Rainer Nögel, Josef Hummelsberger, Stefan N. Willich, Benno Brinkhaus, Michael Teut.
